# Fatal HIV Encephalitis in HIV-Seronegative Patients

**DOI:** 10.3201/eid1501.070834

**Published:** 2009-01

**Authors:** Troy M. Martin, Josiah D. Rich

**Affiliations:** Brown University School of Medicine, Providence, Rhode Island, USA

**Keywords:** HIV encephalitis, seronegative HIV, chronic alcoholism, letter

**To the Editor:** Acute encephalitis is rarely seen in patients infected with HIV (*1*). In addition, HIV in patients who are seronegative is extremely rare, particularly in the setting of current screening ELISAs (*2*). We report a case of encephalitis and HIV in the same patient, which resulted in death.

A 44-year-old Caucasian woman sought treatment at our hospital with a 1-week history of fever, unsteady gait, and progressive confusion. Her medical history included hypothyroidism, depression, and chronic alcohol abuse. The patient’s first tests for HIV were negative at 19 and 12 months prior to admission during routine intake screening for jail inmates (Abbott HIV AB HIV-1/ HIV-2 [rDNA] enzyme immunoassay [EIA] kit; Abbott Laboratories, Abbott Park, IL, USA). Six months before admission, the patient had a viral exanthem of blistering rash on her lips, palate, and chest. Two weeks later, she had oral thrush and a leukocyte count of 1,700 cells/μL. An HIV ELISA result was negative. Three months before admission, she was admitted to a different hospital for weakness, abdominal pain, intermittent fever, diarrhea, persistent oral candidiasis, and ethanol withdrawal. She had leukopenia and thrombocytopenia. A fourth HIV ELISA result was negative. The patient had been admitted to our hospital one week before the current admission with symptoms of fever, confusion, and urinary tract infection. Lumbar puncture showed an elevated protein level (106 mg/dL). A fifth HIV test result 6 days before most recent admission was negative. Five days before admission, she had been discharged to a rehabilitation facility.

On this hospitalization, she had fatigue, headache, disequilibrium, dysarthria, and blurred vision. Initial examination showed fever of 101.3°F, poor word recall, and a wide-based gait. Laboratory tests showed mild anemia and a leukocyte count of 2 × 10^3^ cells/μL.

Over the next few days the patient’s fever persisted and her mental status fluctuated. Tests on hospital day 2 showed a CD4 count of 101/mL (16.9%). Magnetic resonance imaging (MRI) of the brain showed diffuse symmetric white matter disease (Figure, panel A). Samples sent on hospital day 9 eventually showed wild-type HIV with a viral load >500,000 copies/mL. Repeat cerebrospinal fluid (CSF) test results were negative for cryptoccocus antigen, and PCR results were negative for cytomegalovirus, herpes simplex virus (HSV), and JC polyoma virus. The next day, a sixth HIV ELISA result was negative. The serum level of HIV p24 antigen was 202 pg/mL.

**Figure:**
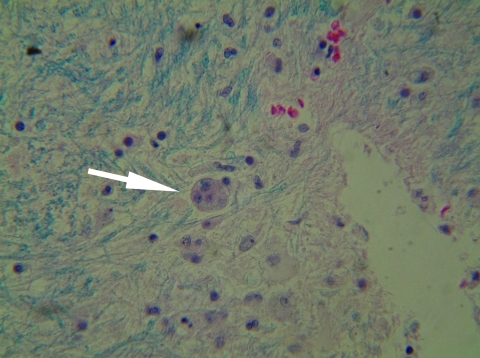
Brain magnetic resonance imaging (MRI) and autopsy findings for the patient. Brain MRI showed diffuse white matter disease on hospital day 2 (A) and marked progression on hospital day 14 (B). Autopsy histopathologic analysis showed microglial nodules, multinucleated giant cells (arrow), perivascular inflammatory cells, vasculopathy with mural fibrosis, perivascular hemosiderin deposition, degeneraton of the central white matter, and neuronal apoptosis (C).

On hospital day 13, the patient began treatment with zidovidine, lamuvidine, didanosine, and nevirapine. Within 24 hours, seizures and catatonia developed in the patient. An electroencephalogram showed diffuse wave form slowing. A repeat MRI showed worsened white matter disease (Figure, panel B). The result of a seventh HIV screening ELISA performed on hospital day 15 was negative. Two days later, the HIV viral load was 241,789 copies/mL. On hospital day 19, her serum levels were within normal limits: immunoglobulin (Ig) M level (164 mg/dL), IgG level (1,440 mg/dL), a 3× normal IgA level (1,060 mg/dL), and no oligoproteins. The CSF had an IgG level >10× normal (72 mg/dL), elevated IgG levels for HSV1 (1:160) and HSV2 (1:40), was negative for virus culture, and showed a negative PCR result for JC polyoma virus. On hospital day 23, the eighth HIV ELISA result was negative. The Abbott HIVAB HIV-1/HIV-2 (rDNA) EIA was used throughout the hospitalization. On hospital day 24, supportive care was withdrawn and the patient died. Throughout her hospitalization, blood, urine, and CSF cultures remained sterile.

Autopsy showed acute HIV encephalopathy and cerebral vasculopathy. The findings included multifocal microglial nodules, perivascular inflammatory cells, vasculopathy with mural fibrosis and perivascular hemosiderin deposition, degeneration of the central white matter, and neuronal apoptosis (Figure, panel C). She also had *Pneumocytis jiroveci* pneumonia and hepatosteatosis without cirrhosis.

There are several possible explanations for the patient’s HIV seroconversion failure. The first explanation is that the patient was subacutely infected but had a retarded humoral response. Delayed seroconversion has been documented up to 42 months after infection (*3*), but this seems unlikely with current ultrasensitive assays. Another possibility is that she was infected with a strain undetectable by screening ELISAs, such as HIV-1 Group N or a rare Group M subtype recombinant variant. This hypothesis also seems unlikely because of the rarity and geographic distribution of these strains (*4*). A third possibility is transient seroconversion with reversion to seronegative status (*5**,**6*). However, given the number and frequency of screening tests in this case, even transient seroconversion would probably have been detected. Another hypothesis, one consistent with the patient’s rapid demise, is infection with a particularly virulent HIV variant, which led to rapid immunocompromise and failure of seroconversion. Such infections have been observed in rapid progressors, in which CD4+ T-cell depletion is so swift that B cells receive no T-cell help and are therefore not able to mount an effective immune response (*7*). In addition, chronic alcoholism may have contributed to immune failure and a rapidly progressive disease course (*8**–**10*).

This case raises several disturbing and interesting questions and possible avenues for future research. The diagnosis of acute HIV encephalopathy with a CD4 count of 100 cells/μL raises the likelihood that this patient was infected with at least 1 strain containing particularly neurotropic properties, possibly with X4 or R5X4 tropism, or that her brain was particularly primed for HIV-induced damage. Understanding the neurotropic properties of different strains of HIV may help prevent similar adverse outcomes in other patients.
